# Indeterminacy of Reverse Engineering of Gene Regulatory Networks: The Curse of Gene Elasticity

**DOI:** 10.1371/journal.pone.0000562

**Published:** 2007-06-27

**Authors:** Arun Krishnan, Alessandro Giuliani, Masaru Tomita

**Affiliations:** 1 Institute for Advanced Biosciences, Keio University, Tsuruoka, Japan; 2 Istituto Superiore di Sanità, Environment and Health Department, Rome, Italy; University of Glasgow, United Kingdom

## Abstract

**Background:**

Gene Regulatory Networks (GRNs) have become a major focus of interest in recent years. A number of reverse engineering approaches have been developed to help uncover the regulatory networks giving rise to the observed gene expression profiles. However, this is an overspecified problem due to the fact that more than one genotype (network wiring) can give rise to the same phenotype. We refer to this phenomenon as “gene elasticity.” In this work, we study the effect of this particular problem on the pure, data-driven inference of gene regulatory networks.

**Methodology:**

We simulated a four-gene network in order to produce “data” (protein levels) that we use in lieu of real experimental data. We then optimized the network connections between the four genes with a view to obtain the original network that gave rise to the data. We did this for two different cases: one in which only the network connections were optimized and the other in which both the network connections as well as the kinetic parameters (given as reaction probabilities in our case) were estimated. We observed that multiple genotypes gave rise to very similar protein levels. Statistical experimentation indicates that it is impossible to differentiate between the different networks on the basis of both equilibrium as well as dynamic data.

**Conclusions:**

We show explicitly that reverse engineering of GRNs from pure expression data is an indeterminate problem. Our results suggest the unsuitability of an inferential, purely data-driven approach for the reverse engineering transcriptional networks in the case of gene regulatory networks displaying a certain level of complexity.

## Introduction

Gene Regulatory Networks (GRNs) have become a major focus of interest in recent years due to the rapid improvement in high-throughput sequencing technologies and advances in computational modeling and information technology. The basic unit of gene regulation consists of a transcription factor, its DNA binding site and the target gene or transcription unit that it regulates [1]. In GRN, transcription factors (TFs) receive inputs from upstream signal transduction processes and in response, bind directly or indirectly, via other TFs or co-factors to target sequences in the promoter or cis-regulatory regions of target genes. These bound TFs can then promote or repress transcription by stimulating or repressing the assembly of preinitiation complexes. The activity of genes is regulated by a host of biological molecules including proteins, peptides and metabolites.

The resulting network is a complex, multilayered system that can be examined at multiple levels of details [2]. The modeling of GRNs has utilized two key approximations [3]. These are: a) control is exercised at the transcriptional level and b) The production of protein product is a continuous process with the rate determined by the balance of gene activation vs. repression. The first constraint, even though it is known to not be tenable in many cases, is considered as a prerequisite while dealing with GRNs. In our approach we relaxed the strict transcriptional character of control by inserting a posttranslational modification (PTM) mechanism into the simulation.

Recent approaches have got rid of the second approximation by including the stochastic nature of production of individual protein molecules. Methods used to model and reverse engineer transcriptional control within gene regulatory systems include the “Boolean” method [4]–[9], the continuous approach using differential equations [10]–[17] that has been well studied and in use for decades and a hybrid Boolean-continuous approach [18], [19]. The interested reader is referred to papers by Smolen et al. [3] and de Jong [20] for a more exhaustive review of the existing approaches.

All the above mentioned reverse engineering approaches have principally focused on decoding the mechanisms of transcriptional control; primarily in order to take advantage of the large amounts of data about RNA transcripts being generated by current genomic technologies. However, measuring peptide, protein and metabolite regulators of gene expression is generally more difficult and not often available [21]. Regardless, in all the techniques mentioned above, one tries to ascertain the genotypic landscape from knowledge of limited phenotypic data. The sense in which genotypes and phenotypes are used in this case is slightly different from their original meaning: genotype points to the underlying functional connectivity between the different gene activities whereas phenotype points to their visible effect (such as mRNA or protein levels). This would have been straightforward if there was a strict one-to-one mapping between genotype and phenotype. However, this is not really the case.

Theoretically, in a given environment, the mapping between any particular genotype and a set of phenotypes is determined by a probability function, which represents a collection of possible phenotypes around the most-likely phenotype for any given genotype [22]. In effect, the genotype to phenotype mapping is bounded by 1:1 mapping (total gene plasticity), 1:all mapping (total gene elasticity) and all:1 mapping (total constraint) with most of the actual cases falling in between these three bounds leading to some degree of what we refer to as “gene elasticity”. This could be due to either decreased environmental canalization or developmental polymorphism or both. Regardless, the fact that multiple genotypes can give rise to a very similar set of phenotypes causes potentially huge problems for the reverse engineering of transcriptional networks.

In this work we study the effect of this particular problem on the inference of gene regulatory networks. We assume that in future (as is already the case with the possibility of the use of large scale protein chips), it would be possible to obtain large-scale information about not just RNA transcripts in a cell but also various protein counts. With that in mind, we simulated a four-gene network in order to produce “data” that we use in lieu of real experimental data. This data was then sampled at a few sampling instants . We then optimized the network connections between the four genes with a view to obtain the original network that gave rise to the data. We did this for two different cases: one in which only the network connections were optimized and the other in which both the network connections as well as the kinetic parameters (given as reaction probabilities in our case) were estimated. We observed that multiple genotypes gave rise to very similar protein levels. Statistical tests indicate that it is impossible to identify different network architectures on the basis of both equilibrium as well as dynamic data. This suggests that a purely data-driven inferential approach for reverse engineering transcriptional networks is improbable if not impossible in practice.

## Materials and Methods

Our approach towards the simulation of GRNs is a mix of the finite-state model pioneered by Brazma et al. [23] and stochastic simulation. The model is based on the following assumptions:

- Each gene has a number of TF binding sites in its promoter region
- Each protein has a number of binding domains, with each binding domain being able to bind to a specific gene.
- The binding of a single activating protein to a binding site creates a complex that can in turn be recognized by RNA Polymerase (RNAP) molecules.
- The binding of a single repressive protein molecule to a binding site creates a complex that can no longer be bound to by RNAP molecules
- An “active” gene is thus denoted by the presence of the corresponding complexes that can be bound to by RNAP molecules.
- Each protein has the possibility of undergoing PTM.
- The PTM can activate or deactivate a protein.

**Figure 1:**
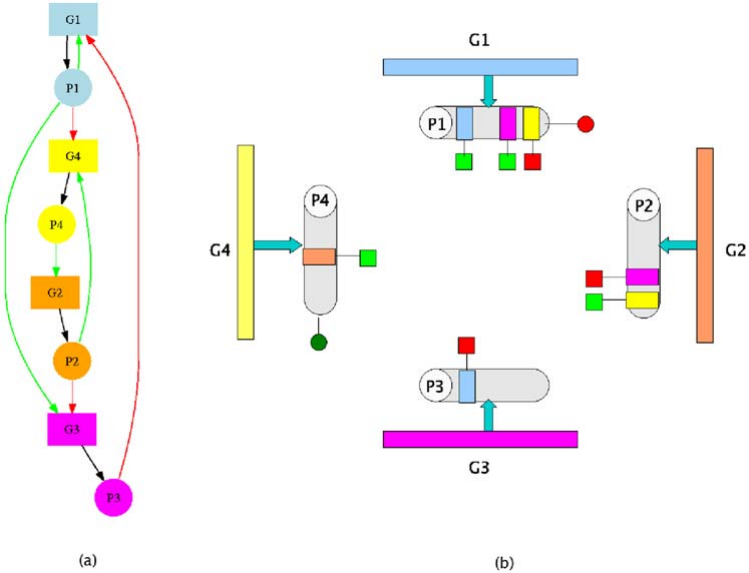
The abstract representation in our model of the network shown in Figure 1(a) is shown in Figure 1(b). The gene corresponding to each protein is represented by different colors. Each protein (colored gray) has a certain number of binding domains. For eg., protein P1 can bind to genes G1, G3 and G4 (showed by the colored bars). The red and green boxes refer to the effect of binding while the red and green circles refer to PTMs.: red represents repression and green, activation

At the basic level, the model can be considered as a finite-state one since the state of the network depends on the binding/unbinding of proteins to the different binding sites in the promoter regions of the different genes. Figure 1(b) shows the abstraction of the network shown in Figure 1(a). The gene corresponding to each protein is colored differently. Each protein has binding domains for none or more genes. As an example, protein P1, has binding domains for genes G1, G3 and G4. The red and green boxes attached to the binding domains indicate the effect of binding: red represents repression while green denotes activation.

A similar abstraction can also be made for the RNAP-cofactor complexes. Each RNAP-cofactor complex can bind to none or more genes in order to transcribe them. The RNAP-cofactor complexes also evolve by either gaining or losing the ability to bind to and transcribe specific genes.

While the genes in Brazma et al.'s model have binary (ON/OFF) states, gene activity in our model is governed by the number of molecules of the “active” gene (that is one with promoter proteins bound to their promoter regions). As a result, the model stays closer to reality where a basal level of gene activity is present and genes are seldom seen to exhibit purely binary state behavior. Additionally, in contrast to the work by Brazma et al. [23], time, in our case is discrete. Moreover, the state affects the number of molecules of each species in the system. Additionally, we also model the effect of reversible PTMs. We describe the model in more detail in the following section.

### Model

Our model of the gene regulatory network involves proteins and DNA molecules interacting in the classical promoter/TF paradigm. This is by no means the unique or the most relevant mechanism of regulatory systems; nevertheless it is endowed with sufficient complexity to be an interesting case study.

Following the work of Hayot et al. [24] and Ingram et al. [25], our model of the gene regulatory network attempts to describe the process of gene regulation from transcription binding to protein production in a physically reasonable way. As mentioned in [25], each gene (*i*) is represented as having a section of DNA (*D_i_*), which codes for the corresponding mRNA (*M_i_*). This is preceded by the binding of transcription factors to the promoter region to form a complex *Q_j_*. The transcription factors are one among the different protein species that are present in the system. The number of proteins in the system usually consists of the inputs to the system as well as of the products of the structural genes in the system. However the protein species can outnumber the genes. This is in order to cater for all types of transcriptional regulators and will be discussed in greater detail below.

RNAP molecules (in combination with other co-factors) can then bind to *Q_i_* as they read the DNA forming a second complex *Q_i_* ^*^. This complex then breaks down on completion of the reading, thereby releasing *Q_i_*, *R_i_* and the newly formed *M_i_*. The mRNA molecules are then translated to produce copies of the protein *P_i_*. Both positive and negative regulations have been included in the model. In case of negative regulation, protein *P_i_* binding to the promoter region of gene *j* will result in the formation of a complex *Q̅_i_*. These molecules cannot be bound to by RNAP-cofactor complex molecules and hence repress the particular gene by inhibiting transcription. The inhibition however is not independent of the binding order. Thus a regulator that inhibits the expression of a gene can only bind a promoter region that has not been already bound by any other transcriptional regulator.

Proteins can also undergo Post Translational Modifications (PTMs). PTMs are of two types: activating and inhibiting. An activating PTM promotes the activity of the protein while an inhibiting PTM deactivates the protein. It must be mentioned that PTMs in our model are reversible. The species *R* can be viewed as either RNAP by itself or as an RNAP-cofactor complex. Typically, in our case, while simulating only the RNAP molecule, a single *R* species was utilized whereas multiple *R* species implied that different RNAP-cofactor complexes were part of the system.

There are 11 species types present in our model as shown in Table 1 while the allowed reactions between these species types are given in Table 2.

**Table 1:** Species present in our model

Species	Description
*I*	Input Proteins (activating signals)
*D*	DNA molecules
*Q*	Transcription factor-DNA complexes (active)
Q*	RNAP-cofactor-Q complexes
*Q̅*	Transcription factor-DNA complexes (inactive)
*R*	RNAP-cofactor complexes
*M*	mRNA molecules
*P*	Protein molecules
P*	Active/Inactive protein molecules for proteins requiring
*T*	PTM agents
*NULL*	NULL molecules for mono-nuclear reactions

**Table 2:** The reactions taking place in our system

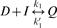
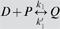
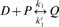
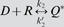
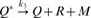
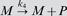
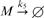
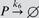
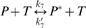

The reactions between a particular section of DNA, *D*, and a protein *P*, or between the complex *Q* and the RNAP-cofactor complex *R* can only take place under certain conditions determined by the type of protein or RNAP-cofactor complex. We model each protein as having potentially up to g DNA-binding domains (one for each gene where g is the number of genes). Similarly, the different types of RNAP-cofactor complex can bind 1, 2, 3, · · ·, *g* DNA-transcription factor complexes *Q*.

There are two different types of transcription factors in the system: Those that influence the expression of other transcriptional regulators but are themselves not transcriptionally regulated and those that do not regulate the expression of other transcriptional regulators. A note must be made here of the fundamental difference between the input proteins (*I* in Table 1) and the normal protein molecules (*P* in Table 1). While both are protein molecules, input proteins act as signaling molecules to the network under study. The input proteins affect the respective *P* molecules in an activating manner. For example, for a 4-node network, if the number of inputs is 2, then *I_1_* will activate *P_1_* and *I_2_* will activate *P_2_*. Moreover, in our simulations, the input proteins are stepped together at time t = 0 and their levels reduced to 0 at time t = 200. Thus these protein levels act as signaling switches.

There are a finite number of PTM-agents (T) and null molecules (NULL) in the system (see Simulation section).

### Simulation

In order to better represent the low copy numbers of all these molecules in the actual cell, we simulate the reactions using a stochastic algorithm. At each time instant, we pick two species at random. We check the compatibility of the species using the reactions given in Table 2. If the two molecules cannot take part in a reaction, say for example *D* and *M,* then no reaction takes place in that time interval. If however, the species can potentially interact, the subtypes of both the species are again chosen at random. If one of the two species is a *P* or an *R* the bits for the corresponding subtype are checked to see that the respective protein or RNAP-cofactor complex can bind to the second species. Additionally, the action of the selected protein species (positive or negative regulatory) is checked to ascertain its effect on the other species. Also, the protein is checked to see if it can undergo PTM and if so whether the PTM is activating or inhibiting. Once all the conditions have been satisfied, a further random number *r* is generated and only if *r*≤*k_i_* where *k_i_* is the probability of occurrence of reaction *i* and the appropriate counts incremented and decreased according to the stoichiometric coefficients given in Table 2. This process is then repeated at the next time interval till the end of the simulation time. Our simulation approach is closest in ethos to that of the StochSim [26] stochastic simulator. We also make use of null molecules in order to simulate monomolecular reactions.

We simulate the model for a total of T time intervals of δt seconds each (with δt = 0.001 for our simulations). A stochastic simulation can give different results depending on the random numbers used. However, in order to obtain “deterministic” results since the goal of this exercise is to ascertain whether the original network can be recovered, we fix the random number seed so that given a particular representation of the proteins and RNAP-cofactor complexes, we will obtain the same, reproducible results. The idea behind using a “deterministic” simulation is to find out whether keeping all other conditions a constant, we are able to get back the original network purely by searching through the space of all possible networks.

### Data

**Figure 2:**
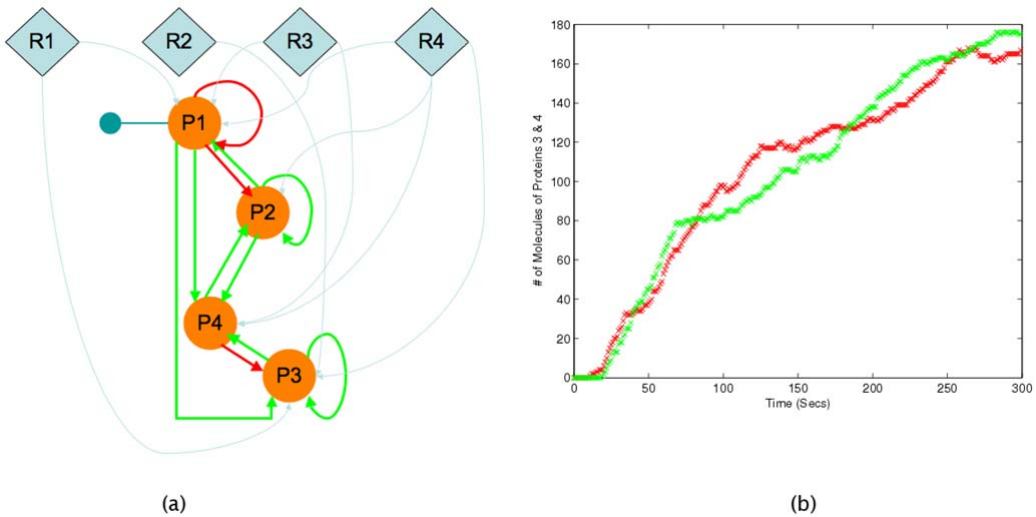
The template network (a) and the protein levels (b) that mimic “experimental data” values.

Figure 2(a) shows the template network that was used to obtain the data that were used in lieu of experimental data. There are four genes in the network with the product of gene 1 requiring an activating PTM in order to regulate the downstream genes. Three of the four genes also auto-regulate themselves; proteins 2 and 3 promote the transcription of their respective genes while protein 1 represses the transcription of its gene. Figure 2(b) show the protein levels for proteins 3 and 4 (with proteins 1 and 2 being almost near zero). The simulation of the template network was carried out for 300 seconds. The data (protein levels of proteins 3 and 4) was sampled at 10-second intervals for a total of 30 data points.

### Network Inference

In order to identify the network that replicates the data, we used a genetic algorithm (GA) that finds the optimum connectivity between the genes and RNAP-cofactor complexes in the system under study. In this case, this equates to finding the best combination of domains in the g proteins and r RNAP-cofactor complexes that results in the minimization of the fitness function. The number of genes, RNAP-cofactor complexes and proteins are denoted by *g*, *r* and *p* respectively. Each protein is represented using *2g+2* bits (also called alleles) while each RNAP-cofactor complex is represented using g bits (alleles), one for each gene. The first *g* bits of each protein represent the binding domains to each of the *g* promoter regions (1 for presence and 0 for absence of the domains), while the next *g* bits indicate the type of regulatory action directed towards the respective proteins (1 for promotion and 0 for inhibition. The last 2 bits represent the effect of PTM, with bit 2*g*+1 representing the presence or absence of PTM and bit 2*g*+2 representing the nature of the PTM (1 for activating or 0 for inhibiting). For example a protein with a representation 1001 − 0001 − 11 (separated by a hyphen for ease of understanding) implies that it can bind to the promoter regions for genes 1 and 4 and that while the regulatory action is negative for gene 1, it is positive for gene 4 (since the first and fourth bits for the second half of the bitstring are 0 and 1 respectively. The protein also requires PTM for activation as suggested by the last two bits (11 implies that PTM is required and it is an activating modification). A similar representation is made for the RNAP-cofactor complex using one bit for each gene. The *p* proteins are encoded using a *(2g+2)p*-long bit string (*2g+2* bits for each protein) and we do the same for the RNAP-cofactor complexes using an *rg*-long bit string. The two encodings are then concatenated to give a chromosome of length *(2g+2)p+rg* bits (alleles). The fitness function to be minimized, *D*(*m, ê*) is given by the root mean squared deviation (RMSD) of the model values *m*, from the “pseudo” experimental values, *é* as: 
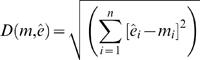
where the number of samples is given by *n*.

Thus in our case, the fitness function itself is more like a “distance” function and will be referred to as such in the rest of the manuscript. As mentioned earlier, a GA was used to find the optimal network acting on the above-described representation. The GA is a stochastic global search method representing a metaphor of natural biological evolution. GAs operate on the population of potential solutions applying the principle of survival of the fittest to produce hopefully, better and better solutions [27]. In each generation, a new set of approximations to the solution is created by a process of selecting individuals according to their level of fitness in the problem domain and breeding them together with operators borrowed from genetics such as crossover, mutations and selections. This process leads to the evolution of populations of individuals (solutions) that are better fitted to the problem domain than their predecessors thereby approaching an optimal solution.

In this case, a population of 500 individuals was seeded. In each generation, two individuals in the population are chosen at random to mate in order to produce offspring. The crossover points in the chromosomes for the mating individuals are based on a certain crossover probability. Mutations can affect chromosomes that are not mating in a given generation with a certain mutation probability (0.8 in our case). Since the GA is a stochastic algorithm, the optimization needs to be done a number of times in order to obtain a ‘near-optimal’ solution. In our case, 25 different runs of the algorithm were carried out and the 6 runs with the smallest distance function values were selected.

The optimization was carried out for two different cases:

- In the first case, the kinetic parameters, represented here by the reaction probabilities, were kept the same as those for the run that generated the data. As a result only the network connections were optimized in order to obtain the network that has the smallest RMSD value.
- In the second case, the search space included the 10 kinetic parameters as well as the network connections.

## Results and Discussion

### Network Inference

**Figure 3:**
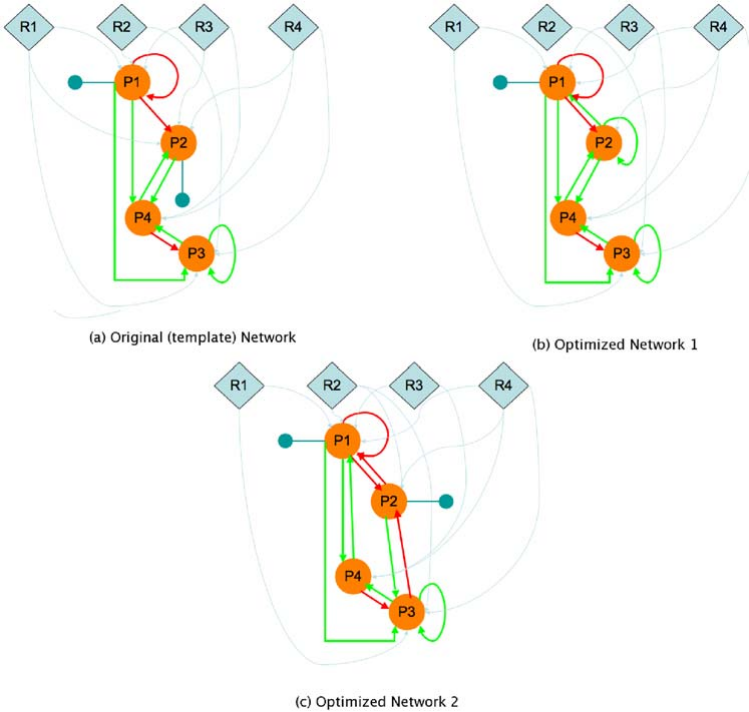
The original (template) network that gave rise to the data and the two best networks obtained from minimizing the error. Only the network connections were optimized in this case, with the kinetic parameters taking on the same values as those that generated the “data”. Both Optimized Network-1 (Figure 3(b)) and Optimized Network-2 (Figure 3(c)) had a value of 0 for the minimization function which was the RMSD of the model and the “data” values.

Figure 3 shows the template network (Figure 3(a)) along with two other networks (Figures 3(b) and 3(c)) that had the smallest distance function values. Interestingly, both the optimized networks had a distance function value of 0; that is, the protein levels at the sampling points were identical to those produced by the template network. While a comparison of the two networks with the template shows some points of similarity, there are also significant differences in the wiring pattern. For example, in both the optimized networks, protein 2 requires an activating PTM in addition to that required by protein 1 whereas it is not so in the original network in which only protein 1 requires an activating PTM. In addition the network in

**Figure 4:**
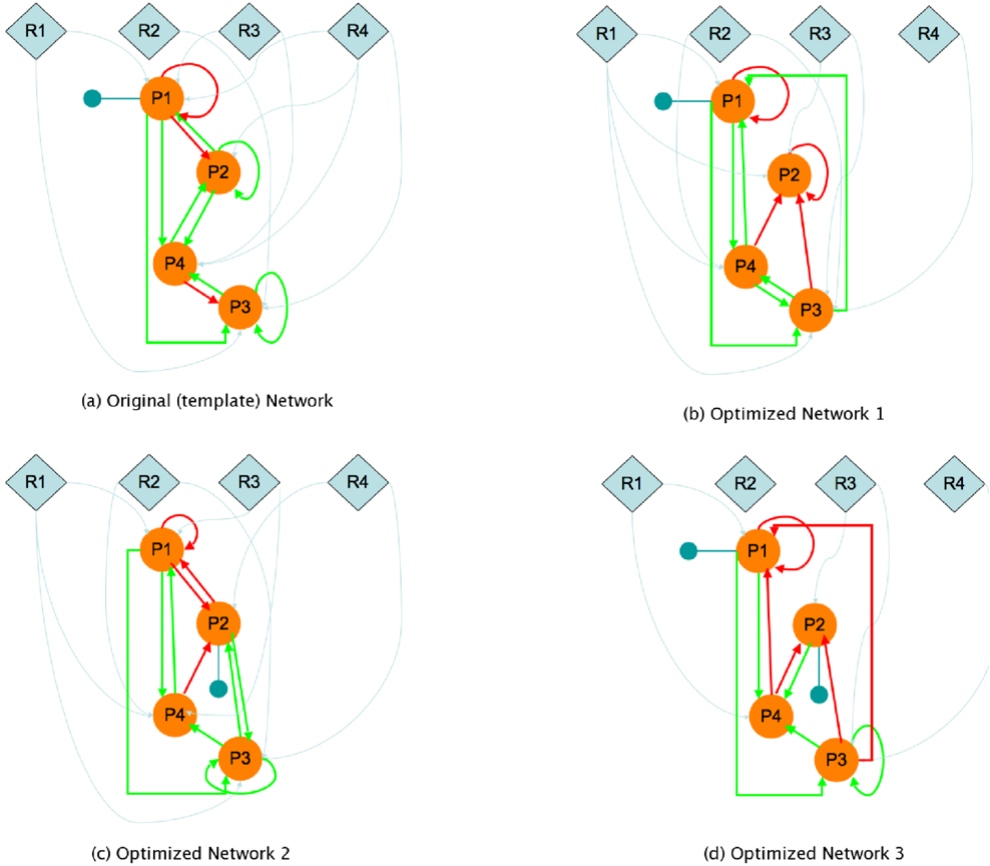
The original (template) network that gave rise to the data and the two best networks obtained from minimizing the error. The network connectivities as well as the reaction probabilities were optimized in order to obtain the minimal deviation between the model and “data” values.

**Figure 5:**
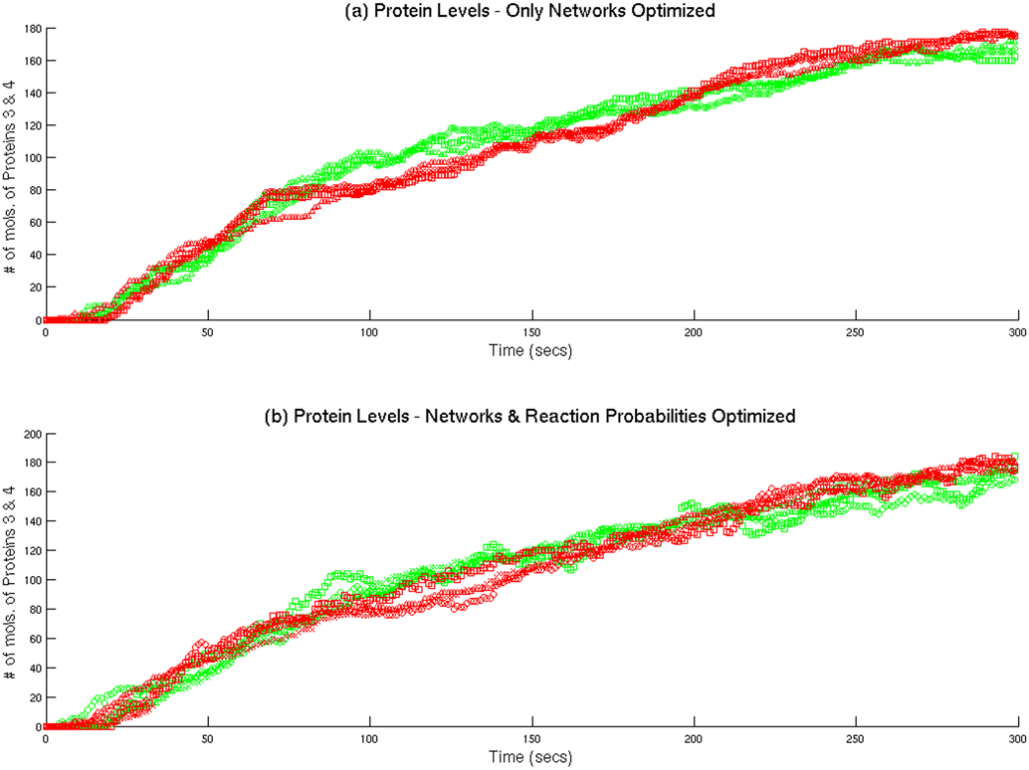
Protein Levels for both experiments: (5(a)): The case where only the network connections are optimized. (5(b)): Case were network connections and reaction probabilities are optimized. Notice that in both cases, the protein expression patterns are very similar despite the differences in network connectivities as seen from Figures 3 and 4.

Figure 3(c) has more repressive interactions than either the template network or the other optimized network in Figure 3(b). As mentioned earlier, the second experiment consisted of identifying the optimal combination of both network connectivities and kinetic parameters that would approximate the data. This is a much tougher optimization problem and hence, despite a number of runs, it was not possible to obtain a solution with an RMSD of 0. Despite this, there were a number of networks that had very similar phenotypes with respect to those produced by the template network. Three of the networks with the smallest RMSD values are shown in Figures 4(b), 4(c) and 4(d). Once again, we witness a wide range of interactions among the elements of the optimized networks, which can be very different from those of the template network. Figures 5(a) and 5(b) show the protein levels obtained by the simulation of six optimized networks with the lowest RMSD values from the two different experiments, respectively.

Despite the wide range of wiring patterns and interaction types (PTM, auto regulation, activation and repression), the protein profiles for all these different networks are remarkably similar to that of the template network shown in Figure 2(b). This is consistent with the fact that there is never total gene-plasticity (a1:1 mapping between genotype and phenotype) but rather, what we call gene “elasticity” with multiple genotypes giving rise to a similar set of phenotypes. This is mainly due to the fact that natural selection acts on variation among phenotypes rather than genotypes [28]. Hence, there is a system of genetic buffering that allows for the buildup and storage of genetic variation in phenotypically normal populations [29]–[31]. Theory suggests that this variation in genotype can change the underlying genetic architecture in spite of the phenotype being maintained due to strong stabilizing selection [32]. This behavior is remarkably similar to what we observe in protein sequence/structure relations (with the sequence playing the role of genotype and the structure of phenotype) where a huge sequence space maps into a much smaller fold space.

### Optimal Networks

**Figure 6:**
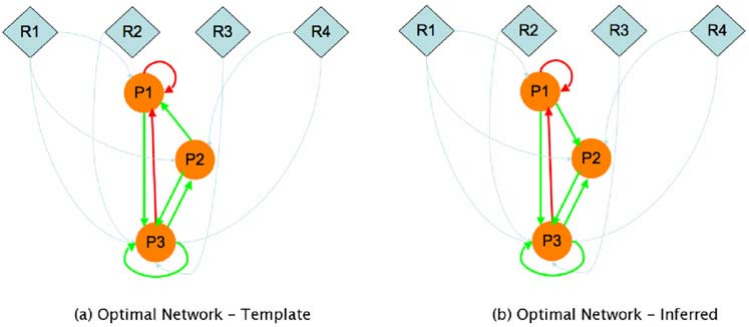
The optimal (template) network and the best network inferred from minimizing the RMSD between the two expression levels. Only the network connectivities were optimized. All other parameters including the kinetic parameters were kept constant. Although the two networks are very similar, there is a slight difference in the activation of P1 and P2.

Although the results in the previous section show that different networks give rise to very similar, even identical expression profiles, a question can be raised as to whether the observed behavior is a result of the fact that the networks might be suboptimal in some sense and hence, the network space surrounding the template network could well be very dense with different networks giving rise to similar expression profiles. This is a valid point and needs to be addressed. However, the notion of optimality itself is difficult to define in this case. What would an “optimal” network look like? We defined an optimal network as one that evolves towards a particular goal from an initial random state. The goal in our case was a maximization of the levels of certain proteins. We evolved a population of networks starting with an initial random assignment towards the goal of maximizing the distance function. Such a network is optimal in terms of the particular distance function. We then performed the same experiment for this “optimal” network by generating data from this network and then using this data to infer back the original network. Figure 6 shows the template and inferred “optimal” networks. As can be observed, the template and optimized networks, although having an RMSD value of 0 (implying perfect alignment of the respective protein expression patterns) do differ slightly in their wiring. Obviously, this is dependent on the particular kinetic parameters used as well as the algorithm being used. However, we would expect to see similar behavior for any stochastic algorithm that models the behavior of gene regulatory networks. Although this is not a conclusive proof of the fact that the behavior observed in the previous section is not completely due to the suboptimality of the template network, it does show that regardless of the type of network, the many-to-one phenotype-genotype mapping can lead to an indeterminacy of the reverse engineering problem.

### Principal Component Analysis

Having demonstrated the equivalence of different network architectures to get rid of a given phenotype and the substantial indeterminacy of reverse engineering problem in the presence of a complex feedback network, we must approach the other horn of the problem. Basically we need to demonstrate that the outputs coming from networks endowed with different architectures cannot be traced back to their respective sources. This step is crucial as proof-of-concept of the basic indeterminacy of the reverse engineering procedure, given that a discrimination of the source networks on the pure basis of their outputs should imply the (at least theoretical) possibility to establish a 1:1 mapping between genotype and phenotype in the presence of sufficient data. For this goal use was made of two different settings correspondent respectively to “equilibrium” and “dynamic” discrimination tasks.

In the first task (equilibrium discrimination) the three networks (R2, R3, template) were run starting from different initial conditions and their occupancy in the different regions of the phase space defined by the “equilibrium” positions of Protein1, Protein3 and Protein4 were examined. As mentioned earlier, the reaching of “equilibrium” was assessed by the stabilization of the protein levels on almost invariant values and in this case was assumed to occur at a time of 300 seconds. Hence from here on, all mention of “equilibrium protein levels” will imply protein levels after a time of 300 seconds.

The different initial conditions were simulated by using different seeds for the random number generator for the different runs. Each network was run for 25 different simulations, each starting from a different initial condition for a total of 300 seconds. Only the protein levels at t = 300 were utilized in this analysis. The reaching of equilibrium was assessed by the stabilization of the protein levels on almost invariant values.

The dynamic discrimination task involved the recording of the different values of Protein1, Protein3 and Protein4 in time during the transient, going from the initial to the stable final position at the end of 300 seconds. The networks were run for 10 different simulations and sampled once every second for a total of 3000 time points. These two tasks correspond to two possible reverse engineering experiments:

1. Discrimination of different mechanisms in space (e.g. different mutations and/or drug treatments)
2. Discrimination of different mechanisms in time (e.g. recording of a time course after a perturbation).

**Figure 7:**
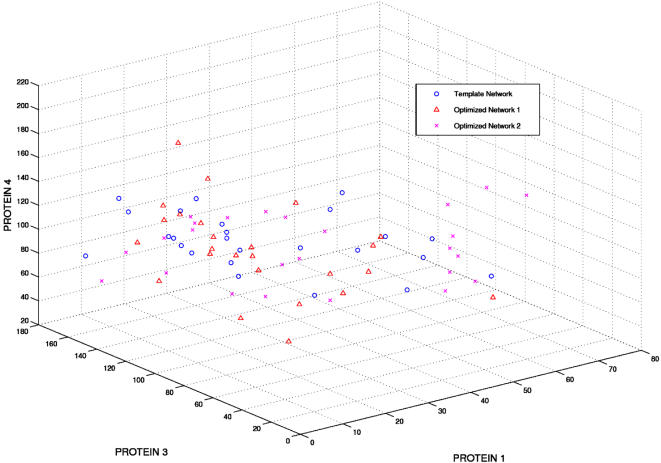
“Equilibrium” phase space for proteins 1, 3 and 4. The reaching of equilibrium was assessed by the stabilization of the protein levels on almost invariant values. In our case, the protein levels at a time corresponding to 300 seconds were assumed to indicate equilibrium levels. There is a lack of a clear separation of the protein levels from the three different networks.

**Figure 8:**
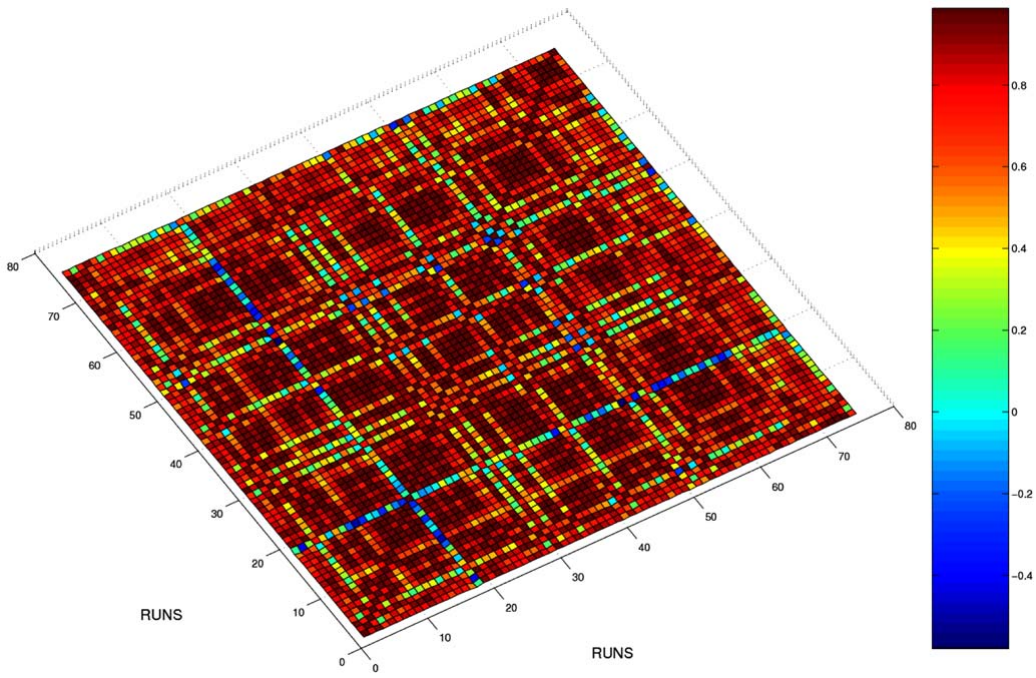
Correlation coefficient of the individual runs for the three different networks against one another. The correlation coefficients are computed as the correlation of the three protein levels for each run and plotted as a matrix with the different runs making up the abscissa and ordinate. Runs 1–25 pertain to the template network, 26–50 to the first optimized network (R2) and runs 51–75 to the second optimized network (R3). The colors range from blue (very low correlation) to dark brown (high correlation). The fact that there is no clear discrimination between the networks implies that the protein levels obtained from the three networks occupy similar regions in phase-space.

The results of the first task are reported in Figure 7. As is evident from the figure, the networks are completely superimposable in the phase space with no discrimination for the phase-space localization. Figure 8 shows the correlation coefficients of the protein levels of each of the 75 different runs (25 runs each for the three different networks) against each other. The first 25 runs are for the template network, followed by those for the R2 and R3 networks respectively. If the networks were indeed separable, then one would expect the correlation coefficient values between runs of the same network to have significantly higher values than between runs from different networks. However, the near uniformity of the high correlation values across networks shows that they do inhabit very similar regions of the phase space.

The dynamic simulation, corresponding to the study of the transient behavior going from an initial (perturbed) state to the attractor (equilibrium protein levels) was analyzed by means of principal component analysis (PCA). The goal of PCA is to project an initial *n*-dimensional space into a *p*-dimensional one (with *p<<n*) saving the major portion of initial information; the new *p* dimensions are called principal components and correspond to linear combinations of the original variables (dimensions) that are orthogonal to each other. The dimensionality reduction is obtained by means of correlations linking the original variables and the components, and correspond to the eigenvectors of the correlation matrix. The portion of the original variance explained by each variable is proportional to the eigenvalue of the corresponding eigenvector [33].

The components correspond to the “order parameters” shaping the data, i.e. to the driving forces generating the observed correlations [34]. A completely random set is expected to give rise to a principal component solution with a flat distribution of eigenvalues. Thus the essential non-random components in a given distribution can be identified as the ones having eigenvalues higher than that expected from pure chance, i.e. above the so called “noise floor” [34].

**Figure 9:**
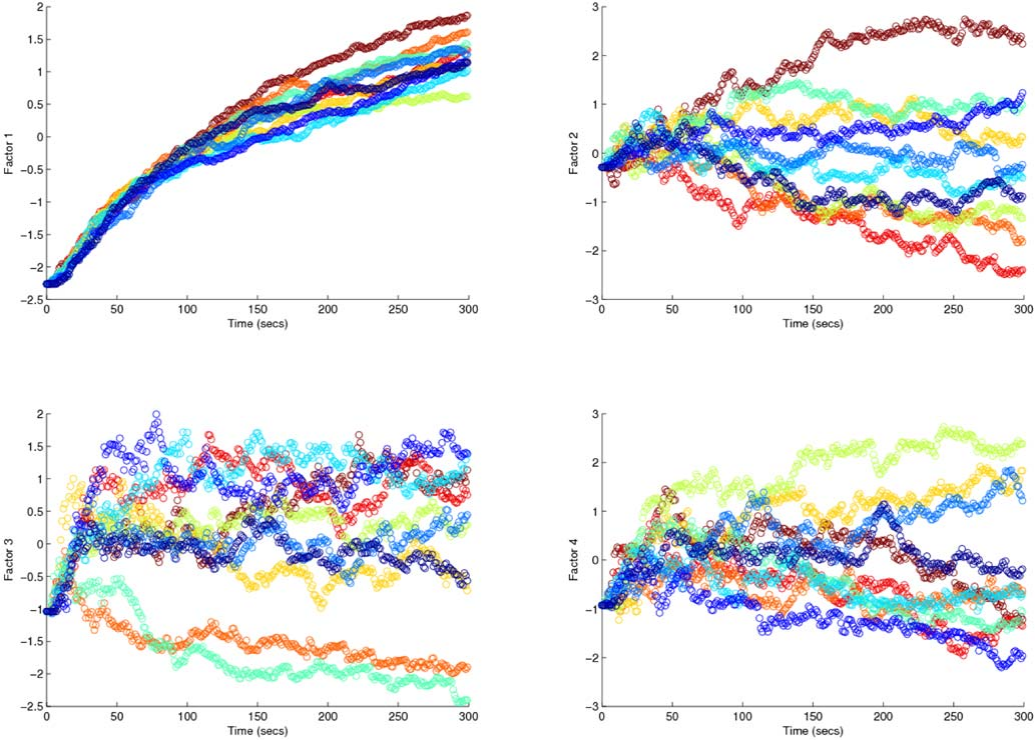
Factor scores for the first four principal components. The different colored lines are the natural separation of the results into the different simulation runs.

In our case each original dimension (variable) had 3000 points corresponding to the 10 time series of 300 points each for the different runs. We had 9 of these variables each corresponding to a specific protein for a specific network (3 protein levels and 3 networks) and the PCA gave rise to a 4-component solution well above the noise floor with a leading first component (factor1) explaining 47.7% of total variance (Table 3). If the networks could be discriminated, one or more of the significant components should be able to separate the statistical units corresponding to the different networks. Looking at the factor-loading matrix reported in Table 4 (the loadings are the correlation coefficients between original variables and components) it is evident that the first factor (component) corresponds to a common “size” component [35] in which all the variables enter with a positive correlation. The presence of such a leading component points to a common behavior relative to both the different networks and different proteins and corresponds to the shape of the curve describing the reach of the attractor (Figure 9, panel a).

**Table 3:** Eigenvalues and the percent of explained variance

	Eigenvalue	Difference	Proportion	Cumulative
1	**4.2960**	**2.6220**	**0.4773**	**0.4773**
2	**1.6739**	**0.6827**	**0.1860**	**0.6633**
3	**0.9913**	**0.2042**	**0.1101**	**0.7735**
4	**0.7871**	**0.2297**	**0.0875**	**0.8609**
5	0.5574	0.2060	0.0619	0.9228
6	0.3514	0.1514	0.0390	0.9619
7	0.1999	0.0911	0.0222	0.9841
8	0.1089	0.0749	0.0121	0.9962
9	0.0341	0.0000	0.0038	1.0000

**Table 4:** Factor Loadings–Correlation coefficients between original variables and components

	Factor 1	Factor 2	Factor 3	Factor 4
R2protein1	0.4308	0.6333	−0.0941	0.3570
R2protein2	0.9217	−0.0551	−0.0525	−0.0239
R2protein3	0.7750	−0.5515	−0.0320	−0.1416
R3protein1	0.5076	−0.6289	0.1555	0.4313
R3protein3	0.5807	0.4311	0.1304	0.4699
R3protein4	0.8781	0.1226	−0.0756	−0.3210
TEMprotein1	0.2231	0.3758	0.8390	−0.2718
TEMprotein3	0.6274	0.4318	−0.4576	−0.2335
TEMprotein44	0.9226	−0.2040	0.1359	−0.0269

The shape components (2 to 4) display both positive and negative loadings and could be responsible for the differences between networks. It is evident from both the loading pattern (Table 4) and component plots (Figure 9 panels b–e) that no component is able to discriminate among the different networks.

From the perspective of the reverse engineering of networks, what this suggests is the improbability, if not the impossibility of inferring GRNs by means of a pure data driven strategy based on the measurement of mRNA or protein levels. The networks obtained from the optimization procedure do show some similarities with the template network. Almost all the networks obtained (with one exception) require protein 1 to have an activating PTM as well as a repressive autoregulatory loop. In addition, all the optimized networks show that protein 1 promotes the transcription of genes 3 and 4. In the first case, where only the network wiring was optimized, it is also discernible that protein 1 inhibits the transcription of gene 2.

However, when both the network and the kinetic parameters were included in the search space, there was no other feature that was as discernible as the ones mentioned above. Given that kinetic parameters are typically unknown and need to be estimated, there is a strong case to be made for the fact that no single network that can be obtained from a completely automated reverse engineering approach can identify the template network that gave rise to the observed phenotype in the first place. Rather, an ensemble of networks can be derived from such approaches. However, even this might not enable us to uniquely determine the underlying network wiring without the additional aid of other data such as those indicating the presence or absence of binding domains for the different proteins, metabolites and other molecules that define the network and so constraining the solution space.

### Conclusions

Bar-Joseph et al. [36] incorporated DNA-protein binding results along with expression profiles in order to describe a genome-wide regulatory network. Since protein-DNA binding data provides direct physical evidence of regulatory interactions, combining genome-wide protein-DNA binding data with gene expression data improves the detection of transcriptional modules over using a single source [37]. The reasoning is that the low data quality and coverage of high-throughput datasets imposes limitations on inferring accurate networks and that technological innovations in data generation and improvements in computational methods will lead to a removal of this roadblock on the path to inferring the underlying network structure accurately. However, we have shown here that even in the presence of completely noise-free data and detailed qualitative models, inferring network connectivities purely from high throughput expression data is almost impossible due to the indeterminacy of the reverse engineering problem. This indeterminacy comes about as a result of gene elasticity with multiple genotypes or network wirings giving rise to very similar, indistinguishable phenotypes.

The impossibility of recovering the exact structure of the network in the presence of feedback loops from input/output relations (definition of a complex machine) was recognized by Heinz von Foerster [38] in the middle of the last century. Thus, the only recourse to accurately uncovering the underlying GRN seems to be to use a combination of data of different origins and scope such as in the work by Bar-Joseph and coworkers.
